# ﻿Two new species of *Drymonia* (Gesneriaceae) from Northwestern South America, including the discovery of the longest flower known in the genus

**DOI:** 10.3897/phytokeys.190.72740

**Published:** 2022-02-17

**Authors:** John L. Clark, Laura Clavijo

**Affiliations:** 1 Science Department, The Lawrenceville School, Lawrenceville, NJ 08648, US A Science Department, The Lawrenceville School Lawrenceville United States of America; 2 Universidad Nacional de Colombia – Sede Bogotá, Facultad de Ciencias, Instituto de Ciencias Naturales, Bogotá, D.C., 111321, Colombia Universidad Nacional de Colombia – Sede Bogotá Bogotá Colombia

**Keywords:** Andes, Chocó biogeographic region, Colombia, Columneinae, *Drymonia*, Ecuador

## Abstract

Two new species, *Drymoniaintermedia* Clavijo & J.L.Clark, **sp. nov.** and *D.longiflora* J.L.Clark & Clavijo, **sp. nov.** (Gesneriaceae, Columneinae), are described from the western Andean slopes of Ecuador and Colombia. The new species are similar to *D.fimbriata*, *D.laciniosa*, *D.macrophylla*, and *D.peponifera* because of the facultative epiphytic habit, deeply serrate to laciniate calyx margins, and fleshy bivalved capsules with tardily dehiscent endocarps. Leaves with brochidodromous venation, narrowly elongate corolla tube, and laciniate calyx margins differentiate *D.intermedia*. The longest corolla of any known *Drymonia* (> 6.5 cm long) differentiates *D.longiflora*. Digital photographs, geographic distributions, and IUCN categories are provided for the new species.

## Abstract (es)

Se describen dos especies nuevas de *Drymonia* (Gesneriaceae, Columneinae) de la vertiente occidental de los Andes ecuatorianos y colombianos: *Drymoniaintermedia* Clavijo & J.L.Clark, **sp. nov.** y *D.longiflora* J.L.Clark & Clavijo, **sp. nov.** Las nuevas especies son similares a *D.fimbriata*, *D.laciniosa*, *D.macrophylla* y *D.peponifera* por ser epífitas facultativas, con márgenes de los lóbulos del cáliz fuertemente aserradas a laciniadas y los frutos en cápsulas carnosas con endocarpos de dehiscencia tardía. *D.intermedia* se diferencia por las hojas con venación broquidódroma, el tubo de la corola estrechamente elongado y los lóbulos del cáliz laciniados. *D.longiflora* se diferencia por tener la corola más larga hasta ahora reportada para el género (> 6.5 cm de largo). Se presentan láminas fotográficas, distribuciones geográficas y categorías de amenaza de la UICN para las especies nuevas.

## ﻿Introduction

The flowering plant family Gesneriaceae, with more than 3400 species and 150+ genera (Weber 2004; Weber et al. 2013), is in the order Lamiales. The family is divided into three subfamilies and seven tribes (Weber et al. 2013, 2020), which represent monophyletic lineages (Ogutcen et al. 2021). The majority of New World members are in the subfamily Gesnerioideae and are represented by 1200+ species and 77 genera (Clark et al. 2020). *Drymonia* Mart. is classified in the tribe Gesnerieae and subtribe Columneinae (Weber et al. 2013, 2020), the largest subtribe with 26+ genera and 16% (ca. 525+ spp.) of the total species’ diversity in the family (Weber et al. 2013, 2020). *Drymonia* is strongly supported as monophyletic based on molecular sequence data (Clark et al. 2015). Our preliminary DNA sequence data strongly support a clade that includes the two species described here: *Drymoniaintermedia* Clavijo & J.L.Clark and *Drymonialongiflora* J.L.Clark & Clavijo. Other members of this clade include *Drymonialaciniosa* Wiehler, *D.macrophylla* (Oerst.) H.E.Moore, and *D.peponifera* J.L.Clark & Clavijo. Digital images are provided to differentiate the two new species from closely related congeners. Table 1 summarizes geographic distributions and diagnostic morphological characters for differentiating *Drymoniafimbriata* C.V.Morton, *D.intermedia*, *D.laciniosa*, *D.longiflora*, *D.macrophylla*, and *D.peponifera*.

**Table 1.** General geographic distribution (names in parentheses indicate provinces for Ecuador or departments for Colombia) and comparison of morphological characters to differentiate *Drymoniafimbriata, D.intermedia, D.laciniosa, D.longiflora, D.macrophylla*, and *D.peponifera*.

	*Drymoniafimbriata* C.V.Morton	*Drymoniaintermedia* Clavijo & J.L.Clark	*Drymonialaciniosa* Wiehler	*Drymonialongiflora* J.L.Clark & Clavijo	*Drymoniamacrophylla* (Oerst.) H.E.Moore	*Drymoniapeponifera* J.L. Clark & L.Clavijo
**Leaf pairs**	isophyllous	isophyllous or subequal	anisophyllous	isophyllous or subequal	isophyllous or subequal	isophyllous or subequal
**Petiole length**	1.5–7.7 cm	0.3–0.6 cm	1.0–2.0 cm	0.7–3.0 cm	0.47–5.24 cm	0.5–3.5 cm
**Leaf venation**	eucamptodromous	brochidodromous	brochidodromous	eucamptodromous	eucamptodromous	eucamptodromous
**Calyx lobe margins**	strongly laciniate with unbranched filiform teeth	laciniate with unbranched filiform teeth	strongly laciniate with branched filiform teeth	deeply serrate to slightly laciniate	serrate to laciniate	deeply serrate to pinnatifid
**Calyx lobe folding**	flat	flat	crispate	flat	flat	crispate
**Corolla length**	2.8–3.5 cm	2.0–3.5 cm	3.0–4.6 cm	6.5–8.0 cm	2–3.5 cm	2–4.5 cm
**Geographic distribution**	Costa Rica & Panama	western Andean slopes in northern Ecuador (Carchi & Esmeraldas)	western Andean slopes in northern Ecuador (Carchi & Esmeraldas)	western Andean slopes in northern Ecuador (Carchi & Esmeraldas) and Colombia (Choco, Valle de Cauca & Nariño); eastern Andean slopes in southern Ecuador (Morona-Santiago)	widespread in Central and South America	eastern Andean slopes of southern Ecuador (Morona-Santiago)

## ﻿Taxonomic treatment

### ﻿ Drymonia intermedia

Taxon classificationPlantaeLepidopteraNotodontidae

Clavijo & J.L.Clark sp. nov.

029B487B-E4A5-59DF-9848-71BF5730D6B0

urn:lsid:ipni.org:names:77254937-1

Fig. 1

#### Diagnosis.

Differs from all *Drymonia* by leaves with a submarginal collecting vein formed by the secondary venation (i.e., brochidodromous), a narrow elongate tubular corolla, and lanceolate calyx lobes with margins laciniate with unbranched filiform serrations. Similar to *D.longiflora*, but differs by smaller corolla tube and smaller calyx lobes. Similar to *D.laciniosa*, but differs by the nearly isophyllous leaves (vs. anisophyllous leaves in *D.laciniosa*).

#### Type.

Ecuador. Esmeraldas: cantón San Lorenzo, parroquia Alto Tambo, trail from the community Durango to Río Tululbi via trail north of highway San Lorenzo-Ibarra, forest managed by Fundación Sirua, 1°2'50"N, 78°36'54"W, 200 m, 28 May 2008, *J.L. Clark*, *B. Bisvicuth & J. Melton III 10344* (holotype: SEL [117454]; isotypes: MO, NY, QCNE, US).

#### Description.

Terrestrial, hemiepiphytic or epiphytic herb or subshrub, with scandent to horizontal shoots to 1 m long. Stems subquadrangular in cross-section, 0.3–0.5 cm in diameter, strigose to glabrate, internodes 2.5–8 cm long. **Leaves** opposite, decussate, usually evenly spaced and becoming clustered near apex, isophyllous or subequal in a pair; petioles 0.3–0.6 cm long, basal enations not evident, strigillose to strigose, terete in cross-section; blade elliptic to ovate, 6.5–12.2 × 2.6–5.5 cm, membranous to subcoriaceous, the base rounded to acute, sometimes oblique, the apex acuminate, the margin serrate, the upper surface glossy-green, glabrescent or strigose, the lower surface light green, glabrescent or strigillose, lateral secondary veins 5–6 pairs, prominent abaxially, strigose abaxially, forming a submarginal collecting vein (i.e., brochidodromous). **Inflorescence** reduced to a single axillary flower in the upper leaf axils; peduncle absent; bracts not observed. **Flowers** with calyx uniformly light green; lobes 5, fused at base, 4 nearly equal, the upper lobe slightly smaller and more narrow, membranous, lanceolate with an elongate acute apex, the margins laciniate with unbranched filiform teeth, the lobes covered with sparsely pilose trichomes, 1.4–1.6 × 0.2–0.3 cm. Corolla zygomorphic, tubular, elongate, 2.0–3.5 cm long, with slight spur (nectar chamber) at base, to 0.4 cm long; the corolla tube perpendicular relative to calyx, ampliate toward the limb, not contracted above, ca. 2.0 cm long, ca. 0.8 cm wide near middle, uniformly pale yellow outside, usually lighter yellow in the throat and limb, sometimes with brown spotting on lower portion of throat, the inside sparsely pilose or glandular, the outside uniformly sericeous; throat to 0.8 cm in diameter; limb with 5 spreading lobes, subequal, globose, rounded at apex, margins fimbriate, uniformly strigose abaxially and adaxially, lobes 3–5 × 4–5 mm, the lower lobe slightly larger. Androecium of 4 didynamous stamens, included; filaments 1.2–2.0 cm long, adnate to the base of the corolla tube for 2–3 mm, glabrous, coiled after anthesis; the anthers at first coherent, after anthesis separating, dehiscent by basal pores, 2–4 × 1.2–1.6 mm. Gynoecium with a single dorsal nectary gland, thick, ovate, 2–3 mm long, glabrous; the ovary superior, sericeous, green; style included, 2–3 cm long, white, puberulent; stigma stomatomorphic. **Fruit** a fleshy bivalved capsule, the valves green abaxially, dark maroon adaxially, at dehiscence reflexed 180°, revealing a central cone of fleshy, dark red funicular tissue covered by a thin, purple endocarp that remains attached and surrounds the placentae and mass of funiculi and seeds, and then dehisces at a later stage. **Seeds** numerous, initially covered by the endocarp, but immersed in the central cone of funicular tissue, each seed 0.4–0.5 × 0.2–0.3 mm, brown, subglobose, pointed at both ends, striate.

#### Phenology.

This species has been collected with flowers in May and June. Specimens with fruits are only known from May.

#### Etymology.

The specific epithet is in reference to the intermediate shapes and sizes of the corolla and calyx between *D.macrophylla*, *D.laciniosa*, and *D.longiflora*.

#### Distribution and preliminary conservation assessment.

*Drymoniaintermedia* is endemic to the western Andean slopes between 150 and 600 m in the Ecuadorian province of Esmeraldas. This species has not yet been found in any formally protected area in Ecuador. According to the IUCN Red List criteria (IUCN 2022) for limited geographic range (EOO <5,000 km^2^ and AOO <500 km^2^) and associated subcriteria, including occurrence at fewer than five locations (B2a) and continuing decline of Andean forests (B2b), *Drymoniaintermedia* should be listed in the category Endangered (EN).

#### Comments.

**Figure 1.**
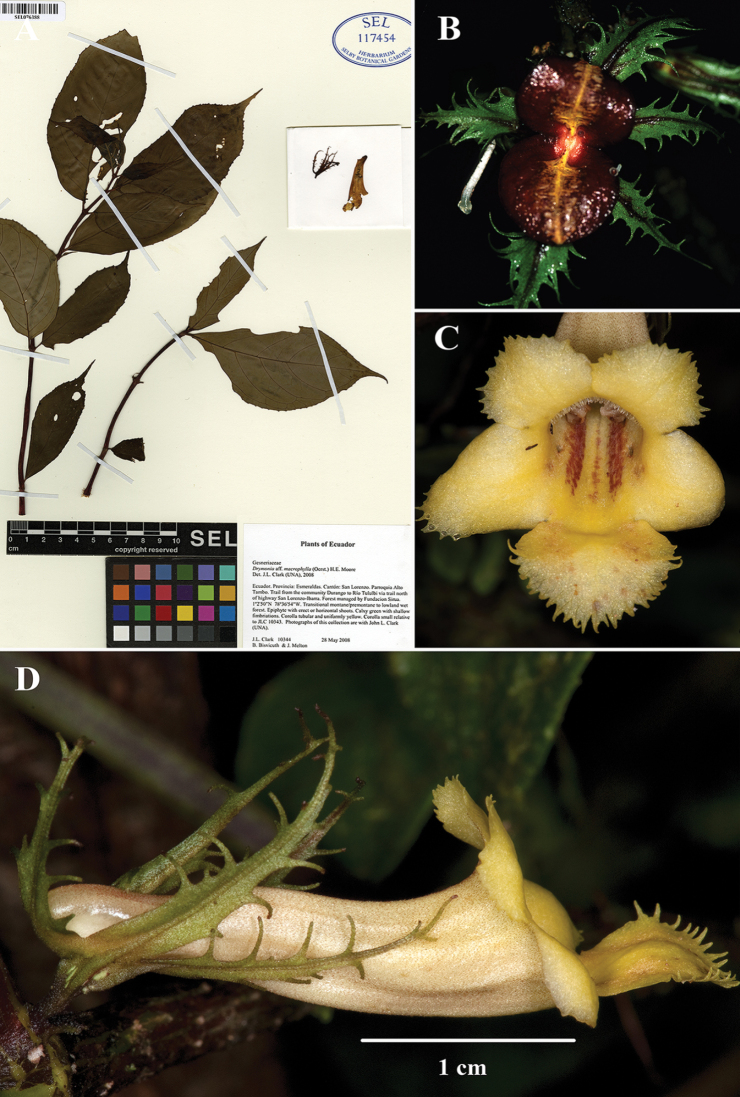
*Drymoniaintermedia* Clavijo & J.L.Clark **A** holotype specimen **B** upper view of mature fruit **C** front view of corolla **D** lateral view of flower (**A** holotype (SEL) of *J.L. Clark et al. 10343***B** from *J.L. Clark et al. 7148***C, D** from *J.L. Clark et al. 10344*). Photos by J.L. Clark.

**Figure 2.**
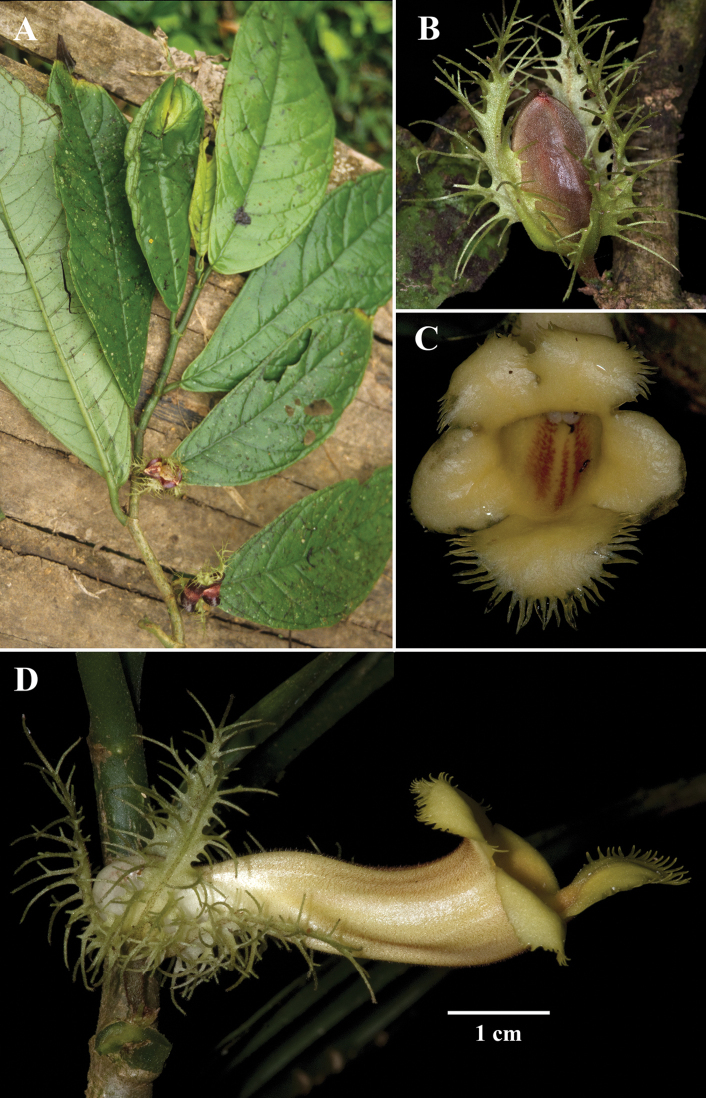
*Drymonialaciniosa* Wiehler **A** dorsiventral habit **B** immature cone-shaped fruit **C** front view of flower **D** lateral view of flower (**A** from *J.L. Clark 1630***B** from *J.L. Clark et al. 9629***C** from *J.L. Clark et al. 10117***D** from *J.L. Clark et al. 12123*). Photos by J.L. Clark.

**Figure 3.**
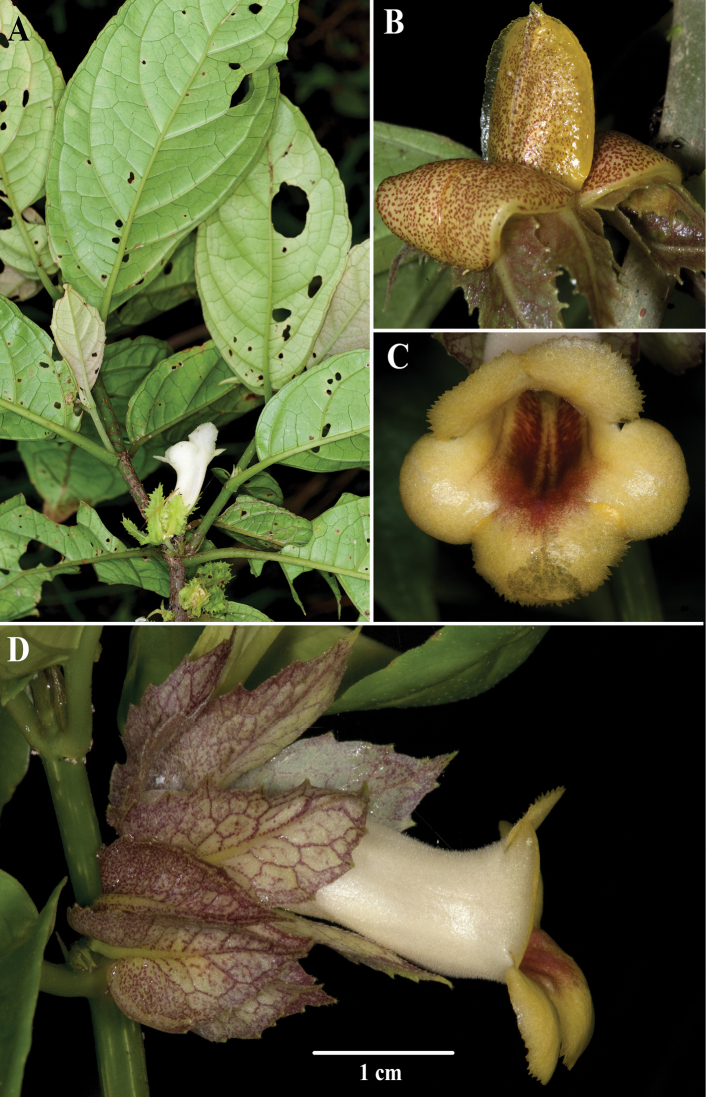
*Drymoniamacrophylla* (Oerst.) H.E.Moore **A** erect subwoody habit **B** mature fleshy bivalved capsule **C** front view of corolla **D** lateral view of flower (**A** from *P. Pedraza et al. 850***B** from *J.L. Clark et al. 12119***C, D** from *J.L. Clark et al. 10044*). Photo **A** by P. Pedraza and photos **B–D** by J.L. Clark.

**Figure 4.**
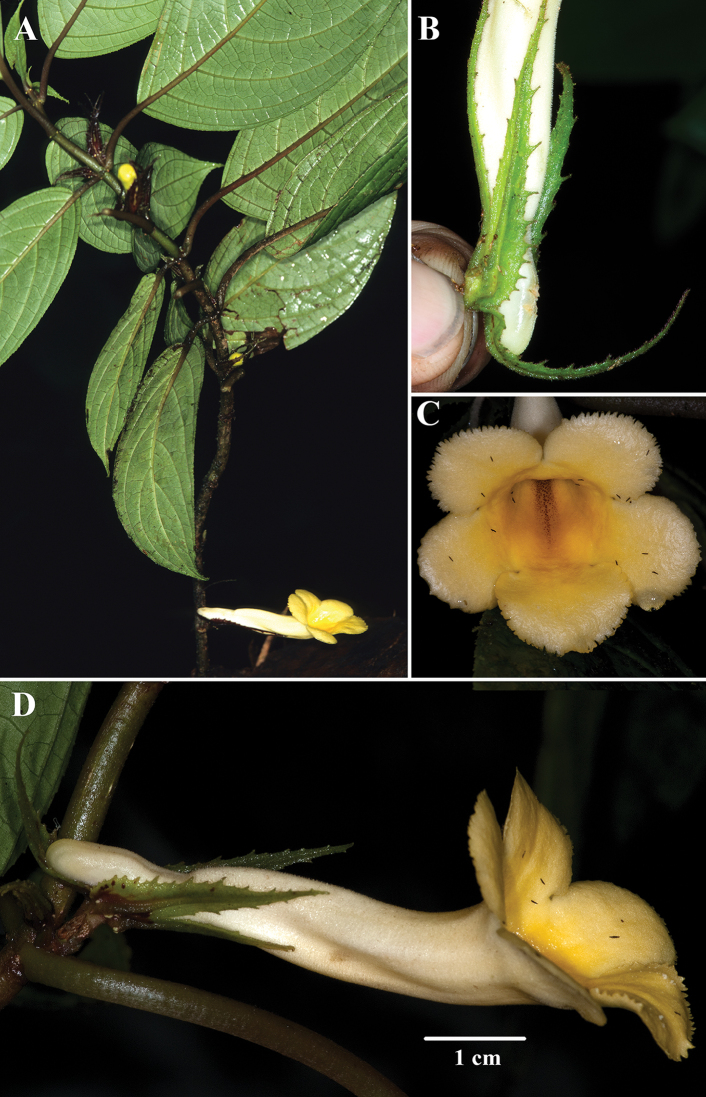
*Drymonialongiflora* J.L.Clark & Clavijo **A** erect subwoody habit **B** mature calyx lobes **C** front view of corolla **D** lateral view of flower (**A** from *J.L. Clark et al. 7196***B** from *J.L. Clark et al. 13417***C, D** from *J.L. Clark et al. 10343*). Photos by J.L. Clark.

The characters that differentiate *D.intermedia* are intermediate between *D.longiflora*, *D.laciniosa*, and *D.macrophylla*. Vegetatively, *D.intermedia* is distinguished by a submarginal collecting vein formed from the secondary veins that curve upwards towards the leaf margin (brochidodromous venation) (Fig. 1A). *Drymonialaciniosa* also has brochidodromous venation, but differs vegetatively by leaves that are anisophyllous (vs. leaves that are nearly isophyllous in *D.intermedia*) (Fig. 2A). Other closely related species, like *D.macrophylla*, have eucamptodromous venation that are defined by secondary veins that curve upwards, but do not form a collecting vein (Fig. 3A). The laciniate calyx lobes of *Drymoniaintermedia* are similar to *D.laciniosa*, but have unbranched filiform serrations (Fig. 1D). In contrast, the calyx lobes of *D.laciniosa* are strongly laciniate with branched filiform serrations (Fig. 2B, D). The calyx lobes shape and margin of *D.intermedia* are similar to *D.longiflora*, but are less than 2 cm long (Fig. 1D). In contrast, the calyx lobes of *D.longiflora* are larger and usually exceed 2.5 cm (Fig. 4D). The corolla in *D.intermedia* is less than 3.5 cm long (Fig. 1D). In contrast, the corolla of *D.longiflora* is greater than 6.5 cm long (Fig. 4D). A summary of diagnostic characters is provided in Table 1.

#### Specimens examined.

**Ecuador. Esmeraldas**: cantón San Lorenzo, parroquia Alto Tambo, border region of Awá Indigenous Territory, entrance to the Río Bogotá community (future biological research station), near Quebrada Pambilar, 0°58'57"N, 78°35'50"W, 350–600 m, 12 Feb 2003, *J.L. Clark*, *G. Zapata & G. Toasa 7148* (MO, QCNE, SEL, US); Esmeraldas: cantón San Lorenzo, parroquia Alto Tambo, trail from the community Durango to Río Tululbi via trail north of highway San Lorenzo-Ibarra, forest managed by Fundación Sirua, 1°2'50"N, 78°36'54"W, 150 m, 2 Jun 2009, *J.L. Clark & The 2009 Gesneriad Research Expedition Participants 11060* (QCNE, SEL, US).

### ﻿ Drymonia longiflora

Taxon classificationPlantaeLepidopteraNotodontidae

J.L.Clark & Clavijo sp. nov.

7B8D6F5A-A0A8-5C00-95BD-A6EB9A0B4075

urn:lsid:ipni.org:names:77254939-1

Fig. 4

#### Diagnosis.

Differs from all *Drymonia* by an elongate corolla that exceeds 6.5 cm in length, the longest corolla known in the genus. The subshrub habit with elongate shoots and corolla shape are similar to *Drymoniamacrophylla* and *D.peponifera. Drymoniamacrophylla* has a corolla that rarely exceeds 3.5 cm in length and *D.peponifera* has a corolla that rarely exceeds 4.5 cm in length.

#### Type.

Ecuador. Esmeraldas: cantón San Lorenzo, parroquia Alto Tambo, remnant patch of primary forest on north side of road between Durango and Alto Tambo on highway San Lorenzo-Ibarra, 0°57'19"N, 78°33'30"W, 695 m, 29 May 2008, *J.L. Clark*, *B. Bisvicuth*, *S. Ginzbarg & J. Melton III 10442* (holotype: US; isotypes: COL, E, G, MO, NY, QCNE, SEL).

#### Description.

Terrestrial, hemiepiphytic or epiphytic herb or subshrub, with scandent to horizontal shoots to 1 m long. Stems subquadrangular in cross-section, 0.3–0.6 cm in diameter, strigose to glabrate, internodes 1.0–8 cm long. **Leaves** opposite, decussate, usually evenly spaced and becoming clustered near apex, subequal to unequal in a pair; petioles 0.7–3.0 cm long, basal enations present, strigillose to strigose, terete in cross-section; blade elliptic to ovate, 8.0–28 × 3.5–13 cm, membranous to subcoriaceous, the base rounded to acute, sometimes oblique, the apex acute to long-acuminate, the margin serrulate, the upper surface glossy-green, glabrescent or strigose, the lower surface light green with red venation, glabrescent or strigillose, lateral secondary veins 5–7 pairs, prominent abaxially, strigose abaxially. **Inflorescence** reduced to a single flower in the upper or lower leaf axils; peduncle absent; bracts small, lanceolate, 3.0–7.0 × 1.1–1.3 mm, green, lanceolate, strigose; pedicels short, 3–7 mm long, green, strigose. **Flowers** with calyx green, dark red with green margins, or uniformly dark red, with enations at base; lobes 5, nearly free, 4 equal, the upper lobe slightly smaller and more narrow, membranous, lanceolate with an elongate acute apex, the margins deeply serrate to slightly laciniate, lobes covered with sparsely pilose trichomes, 1.5–2.7 × 0.3–0.5 cm. Corolla zygomorphic, tubular, elongate, 6.5–8.0 cm long, with slight spur (nectar chamber) at base, 0.5–0.7 cm long; the corolla tube perpendicular relative to calyx, ampliate toward the limb, not contracted above, 5.0–6.5 cm long, 0.5–1.1 cm wide at the mid portion, uniformly pale yellow and sericeous outside, usually darker yellow inside; throat to 1.4 cm in diameter, the inside yellow, sometimes with brown spotting on lower portion, sparsely pilose or glandular; limb with 5 spreading lobes, subequal, globose, rounded at apex, margins erose, glabrous abaxially and adaxially, lobes 5–10 × 4–11 mm, the lower lobe slightly larger. Androecium of 4 didynamous stamens, included; filaments 5–6 cm long, adnate to the base of the corolla tube for 2–3 mm, glabrous, coiled after anthesis; the anthers at first coherent, after anthesis separating, dehiscent by basal pores, 3–5 × 2–1.8 mm. Gynoecium with a single dorsal nectary gland, thick, ovate, 2–3 mm long, glabrous; the ovary superior, sericeous, green; style included, 5–6 cm long, white, puberulent; stigma stomatomorphic. **Fruit** and seeds not observed.

#### Phenology.

This species has been found with flowers in two periods: February to May and August to October.

#### Etymology.

The specific epithet is in reference to the elongate corolla tube, the longest of any known species of *Drymonia*.

#### Distribution and preliminary conservation assessment.

*Drymonialongiflora* is locally abundant in forests along the western slopes of the northern Andes in Ecuador (Provinces Carchi and Esmeraldas) and Colombia (Departments Chocó, Valle del Cauca, and Nariño). One disjunct population is documented from southern Ecuador (Province Morona-Santiago). *Drymonialongiflora* grows in mature shaded forests, from 150 to 900 m in elevation. We provisionally assess this new species as Vulnerable (VU), according to the IUCN Red List criteria (IUCN 2022) for limited geographic range (EOO < 20,000 km^2^ and AOO <2,000 km^2^) and associated subcriteria, including occurrence at fewer than ten locations (B2a) and continuing decline of Andean forests (B2b).

#### Comments.

**Figure 5.**
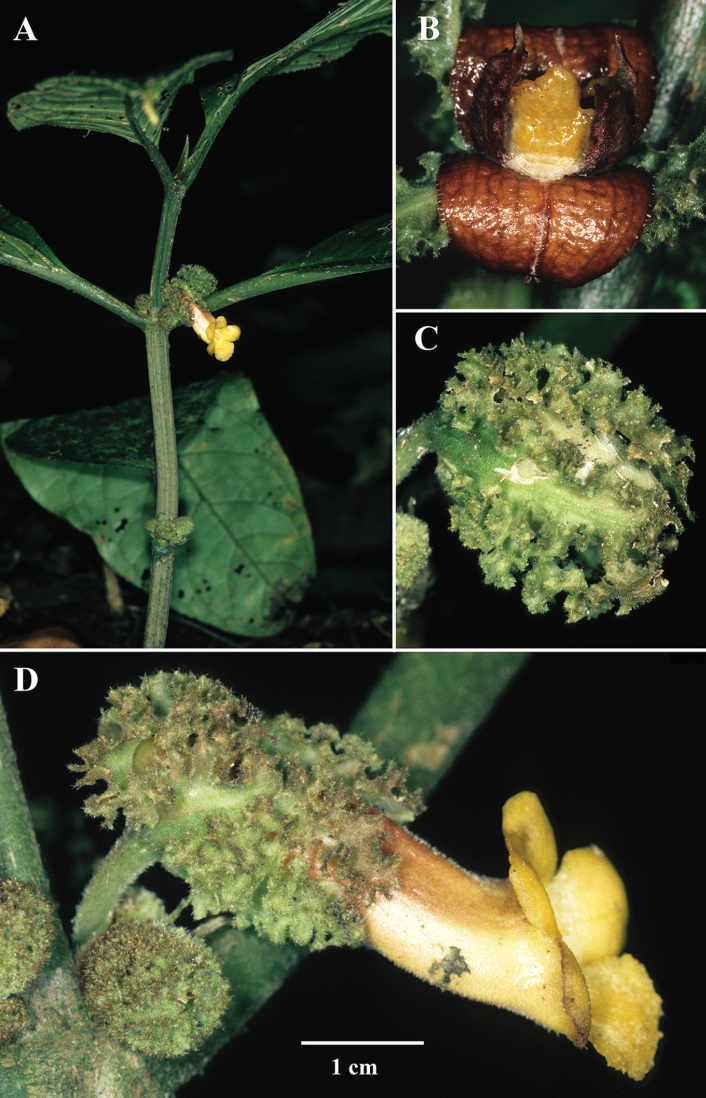
*Drymoniapeponifera* J.L.Clark & Clavijo **A** habit **B** upper view of mature fruit **C** lateral view of immature calyx lobes **D** lateral view of axillary inflorescence with a single mature flower (**A–D** from *J.L. Clark & L. Jost 6957*). Photos by J.L. Clark.

*Drymonialongiflora* is distinguished by the longest known corolla recorded for *Drymonia* (> 6.5 cm). The unbranched subwoody epiphytic or terrestrial subshrub habit and the foliage are similar to *Drymoniamacrophylla*, *D.fimbriata*, and *D.peponifera*. All four species are vegetatively similar, but readily differentiated by corolla tube length and calyx lobes (Fig. 5C, D). The calyx lobes of *Drymoniapeponifera* appear crispate from the reflexed and undulating lobes (Fig. 5C, D). In contrast, the calyx lobes of *D.macrophylla* (Fig. 3D) and *D.longiflora* (Fig. 4D) are flat. The margins of the calyx lobes in *Drymonialongiflora* are serrate to slightly laciniate, in contrast to the strongly laciniate with unbranched filiform teeth of *D.fimbriata*. The corolla in *Drymonialongiflora* exceeds 6.5 cm in length (Fig. 4D). In contrast, the corolla rarely exceeds 3.5 cm in *D.macrophylla* (Fig. 3D) and *D.fimbriata*. The corolla is less than 4.5 cm in *D.peponifera* (Fig. 5D). *Drymonialongiflora* and *D.intermedia* share similar shapes for corolla tubes and calyx lobes, but differ in size, with *D.intermedia* much smaller. A summary of diagnostic characters is provided in Table 1.

Our preliminary DNA sequence data strongly support a close relationship in a clade that includes *D.intermedia*, *D.laciniosa*, *D.longiflora*, *D.macrophylla*, and *D.peponifera*. This clade is defined by fleshy capsular fruits with tardily dehiscent endocarps. A photographic guide and description of fruit types is summarized in Clark and Clavijo (2022). Most *Drymonia* capsules have endocarps that dehisce when mature. In contrast, the clade that includes *D.intermedia* and *D.longiflora* is defined by endocarps that remain attached and surround the placentae and mass of funiculi and seeds (Figs 1B, 5B). The endocarp eventually becomes dehiscent at a later stage when it detaches from the reflexed outer layers of the fruit wall (Fig. 5B). Although we have not observed mature fruits of *Drymonialongiflora*, we predict that the fruit type of this species shares the same characters with closely related congeners.

#### Specimens examined.

**Colombia. Chocó**: 2 km south of Las Animas on road to Istmina, 150 m, 13 Aug 1976, *A. Gentry & M. Fallen 17620* (COL); **Valle del Cauca**: municipio Buenaventura, carretera vieja a Benaventura, 900–1500 m Apr 1998, *M. Amaya-M. & J.F. Smith 594* (COL, US); municipio Buenaventura, Los Tubos, Pericos, vertiente occidental de cordillera occidental, km 43 Cali-Buenaventura, 3°51'00"N, 76°47'19"W, 521 m, 30 Aug 2011, *J. Home 228* (CUVC); Bajo Calima, concesión Pulpapel/Buenaventura, carretera a San Isidro, 3°55'N, 77°W, 100 m, 30 Sep 1987, *M. Monsalve B. 1865* (CUVC, MO); road Cali-Buenaventura, further down the road towards Buenaventura, about 4 km, 1 May 1972, *H. Wiehler*, *R.L. Dressler*, *N.H. Williams & N.F. Williams 72106* (SEL); municipio Dagua, Vereda Yatacue, sitio La Riqueza, camino desde el tunel a las torres, 3°35'40.6"N, 76°53'31.8"W. 630–780 m, 20 Jan 2019, *L. Clavijo*, *M. Perret*, *K. Arango*, *A. Zuluaga & W. Villar 2231* (COL, CUVC, G). **Nariño**: municipio Barbacoas, corregimiento El Diviso, vereda El Verde, western slopes of the Cordillera Occidental, remnant forest along highway between Altaquer and El Diviso, 1°21'46"N, 78°10'32"W, 795 m, 13 May 2013, *J.L. Clark*, *L. Clavijo*, *O. Marín & M. Flores 13417* (COL, CUVC, SEL, US); municipio de Ricaurte, Resguardo Indígena Nulpe Medio, camino a la quebrada La Conga, 1°6'N, 78°13'W, 750 m, 8 Jan 1996, *M.S. González & B.R. Ramírez-P. 1606* (PSO); municipio de Ricaurte, Resguardo Indigena Nulpe Medio Andalucía, quebrada La Babosa, 1°5'N, 78°14'W, 780–815 m, 12 Jan 1996, *M.S. González & B.R. Ramírez-P. 1678* (PSO). **Ecuador. Carchi**: border area between Carchi and Esmeraldas, about 30 km past Lita on road Lita-Alto Tambo, 450 m, 28 Jun 1991, *H. van der Werff*, *B. Gray & G. Tipas 12113* (MO, QCNE, SEL, US). **Esmeraldas**: cantón San Lorenzo, parroquia Alta Tambo, Awá Indigenous Territory, Río Bogotá community (future biological research station), 2 km S of Lita-San Lorenzo road, near Quebrada Pambilar, 0°58'57"N, 78°35'60"W, 350–600 m, 12 Feb 2003, *J.L. Clark*, *G. Zapata & G. Toasa 7129* (MO, QCNE, SEL, UNA); cantón San Lorenzo, parroquia Alta Tambo, border region of Awá Indigenous Territory, entrance to the Río Bogotá community (future biological research station), near Quebrada Pambilar, 0°58'57"N, 78°35'50"W, 350–600 m, 13 Feb 2003, *J.L. Clark 7197* (F, MO, QCNE, SEL, UNA, US); parroquia Alto Tambo, trail from the community Durango to Río Tululbi via trail north of San Lorenzo-Ibarra highway, forest managed by Fundacion Sirua, 1°2'50"N, 78°36'54"W, 150–200 m, 28 May 2008, *J.L. Clark*, *B. Bisvicuth & J. Melton III 10344* (MO, NY, QCNE, SEL, US); cantón San Lorenzo, remnant patch of forest along the Ibarra-San Lorenzo highway, between Durango and Alto Tambo, 1°0'33"N, 78°35'58"W, 516 m, 3 Jun 2009, *J.L. Clark J.L. Clark & The 2009 Gesneriad Research Expedition Participants 11113* (MO, NY, QCNE, SEL, US); cantón San Lorenzo, remnant patch of forest along highway Ibarra - San Lorenzo, between Durango and Alto Tambo, 1°0'33"N, 78°35'58"W, 516 m, *J.L. Clark & The 2009 Gesneriad Research Expedition Participants 11127* (QCNE, SEL, US); cantón Eloy Alfaro, Reserva Ecológica Cotachi-Cayapas, parroquia Luis Vargas Torres, Río Santiago, 0°49'S, 78°45'W, 250 m, 8 Dec 1993, *M. Tirado*, *P. Asimbaya*, *M.I. Corosa & V. Arroyo 776* (MO, QCNE, US); cantón San Lorenzo, San Francisco, recinto Durango, sector Colinado, terrenos propiedad Sr. Demetrio Paez, 1 km al este de la carretera Lita-San Lorenzo, 1°2'N, 78°36'W, 256 m, 17 Oct 1999, *J.C. Valenzuela & E. Freire 474* (QCNE, MO, US). **Morona-Santiago**: Región de la Cordillera del Cóndor y Cordillera de Cutucú, Cordillera de Shaime, al norte del Río Santiago, Centro Shuar Jempekat, 2°57'S, 77°50'W, 600 m, 15 Oct 2003, *G. Toasa 9343* (MO, QCNE).

## Supplementary Material

XML Treatment for Drymonia intermedia

XML Treatment for Drymonia longiflora

## References

[B1] Clark JL Clavijo L Muchhala N (2015) Convergence of anti-bee pollination mechanisms in the Neotropical plant genus *Drymonia* (Gesneriaceae). Evolutionary Ecology 29: 355–377. 10.1007/s10682-014-9729-4

[B2] Clark JL Clavijo L (2022) *Drymoniapeponifera*, a new species of Gesneriaceae from Ecuador with an overview of *Drymonia* fruit traits. Brittonia. 10.1007/s12228-021-09688-3

[B3] Clark JL Skog LE Boggan JK Ginzbarg S (2020) Index to names of New World members of the Gesneriaceae (Subfamilies Sanangoideae and Gesnerioideae). Rheedea 30: 190–256. 10.22244/rheedea.2020.30.01.14

[B4] IUCN (2022) Guidelines for Using the IUCN Red List Categories and Criteria. Version 15. Prepared by the Standards and Petitions Committee. [accessed 20.01.2022]

[B5] Ogutcen E Christe D Nishii K Salamin N Möller M Perret M (2021) Phylogenomics of Gesneriaceae using targeted capture of nuclear genes. Molecular Phylogenetics and Evolution 157: e107068. 10.1016/j.ympev.2021.107068 33422648

[B6] Weber A (2004) Gesneriaceae. In: Kadereit J (Ed.) The Families and Genera of Vascular Plants.Vol. 7. Flowering Plants. Dicotyledons. Lamiales (Except Acanthaceae Including Avicenniaceae). Springer, Berlin, 63–158. 10.1007/978-3-642-18617-2_8

[B7] Weber A Clark JL Möller M (2013) A New Formal Classification of Gesneriaceae. Selbyana 31(2): 68–94.

[B8] Weber A Middleton DJ Clark JL Möller M (2020) Keys to the infrafamilial taxa and genera of Gesneriaceae. Rheedea 30: 5–47. 10.22244/rheedea.2020.30.01.02

